# The Implications of Liver Biopsy Results in Patients with Myeloproliferative Neoplasms Being Treated with Ruxolitinib

**DOI:** 10.1155/2019/3294046

**Published:** 2019-01-06

**Authors:** Douglas Tremblay, Juan Putra, Alexander Vogel, Adam Winters, Ronald Hoffman, Thomas D. Schiano, Maria Isabel Fiel, John O. Mascarenhas

**Affiliations:** ^1^Tisch Cancer Institute, Icahn School of Medicine at Mount Sinai, New York, NY, USA; ^2^Department of Pathology, Icahn School of Medicine at Mount Sinai, New York, NY, USA; ^3^Department of Medicine, Icahn School of Medicine at Mount Sinai, New York, NY, USA; ^4^Department of Medicine, Division of Liver Diseases, Recanati/Miller Transplantation Institute, Icahn School of Medicine at Mount Sinai, New York, NY, USA

## Abstract

Ruxolitinib is increasingly being utilized for the treatment of myelofibrosis and polycythemia vera, but the potential for hepatic toxicity is poorly understood. We performed a retrospective review of hepatic damage occurring in patients with myeloproliferative neoplasms receiving ruxolitinib. Relevant histologic images of liver biopsies were reviewed by an experienced liver pathologist and reported to a multidisciplinary team including hepatology and hematology. A variety of liver pathology was observed including extramedullary hematopoiesis, obliterative portal venopathy, and drug-induced liver injury. In all cases reviewed, the liver biopsy had significant treatment implications. We conclude that hepatology referral and liver biopsy in patients receiving ruxolitinib therapy with biochemical evidence of liver injury reveals a variety of etiologies which have significant treatment impact. Clinicians should be aware of the potential causes of liver damage in this population and initiate prompt referral and liver biopsy.

## 1. Introduction

Ruxolitinib is a JAK1/2 inhibitor and is the sole FDA-approved therapy for patients with intermediate-/high-risk myelofibrosis (MF), and it is also approved to treat patients with polycythemia vera (PV) that have previously failed therapy with hydroxyurea. Ruxolitinib treatment has been associated with transient mild aminotransferase elevations during preapproval clinical trials, with grade 1, by Common Terminology Criteria for Adverse Event (CTCAEs), alanine aminotransferase (ALT), and aspartate aminotransferase (AST) elevations seen in 25.2% and 17.4% of patients, respectively [1]. Another estimate based on preapproval regulatory reviews cites the incidence of aminotransferase elevations as 18% in those receiving ruxolitinib [2]. No cases of acute hepatic failure have been reported to date.

MF itself is also associated with hepatic dysfunction. Examples include invasion of hepatic tissue with hematopoietic cells in the setting of extramedullary hematopoiesis (EMH), portal vein thrombosis, and obliterative portal venopathy (OPV) [3]. EMH in particular is important to identify as it is a disease manifestation that may respond favorably to ruxolitinib therapy [4]. With the increasingly widespread use of ruxolitinib in the treatment of MF and PV patients, clinicians may be faced with the dilemma as to whether ruxolitinib is the cause of hepatic enzyme elevations or is in fact beneficial for the treatment of hepatic dysfunction arising from EMH. Herein, we describe a series of patients without known liver disease or pathology receiving ruxolitinib who experienced hepatocellular damage and had a liver biopsy performed that assisted in their subsequent management.

## 2. Cases

### 2.1. Case 1

**Figure 1:**
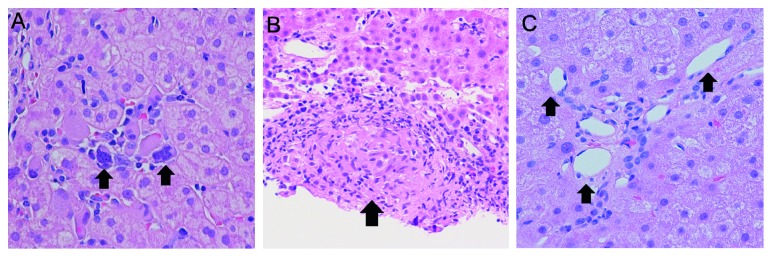
(A) The liver biopsy specimen shows prominent extramedullary hematopoiesis in the sinusoids. Megakaryocytes are marked by the arrows. (B) An epithelioid granuloma (arrow) is noted in the portal tract. An adjacent area shows sinusoidal dilatation. (C) A portal tract with herniated portal veins(arrows). The findings are compatible with obliterative portal venopathy.

A 55-year-old male with a history of *CALR*-positive, low-risk MF by the Dynamic International Prognostic Scoring System (DIPSS) was initiated on 5 mg twice daily ruxolitinib treatment given progressive splenomegaly and worsening night sweats. He experienced an outstanding symptomatic response without significant improvement in splenomegaly. His aminotransferases, which were normal prior to ruxolitinib initiation, became mildly elevated, with ALT rising from 64 U/L at initiation to 232 U/L after 5 months of therapy. The patient was not on other hepatotoxic medications. A transjugular liver biopsy was obtained, which demonstrated significant EMH and diffuse sinusoidal infiltration with atypical appearing megakaryocytes, without evidence of steatohepatitis or drug-induced liver injury (DILI) (Figure 1(A)). Given the finding of EMH, the ruxolitinib dose was increased to 10 mg twice daily with immediate and sustained improvement in ALT to 85 U/L. He is currently being evaluated for an allogeneic hematopoietic stem cell transplantation.

### 2.2. Case 2

A 66-year-old male with a history of PV was initiated on ruxolitinib 10 mg twice daily for worsening leukocytosis and massive splenomegaly. He experienced an excellent initial response with significant reduction in palpable splenomegaly by 50%. However, serum levels of alkaline phosphatase (ALP) began to rise from a baseline of 113 U/L to 311 U/L after 2 weeks of exposure to drug. The ALP peaked at 1286 U/L after approximately 8 months of ruxolitinib exposure. He did not start any other medications or supplements during this time. He was continued on a higher dose of ruxolitinib at 15 mg twice daily for presumed EMH. The ALP remained elevated at 334 U/L, so a liver biopsy was performed at that time, demonstrating granulomatous hepatitis with ductopenia (Figure 1(B)), which was attributed to DILI. Shortly afterwards, he expired from hypoxemic respiratory failure in the setting of a lobar pneumonia. This represents a potential case of DILI due to ruxolitinib given the temporal relationship between ruxolitinib initiation and a grade 3 ALP rise, and further supported by the liver biopsy findings.

### 2.3. Case 3

A 74-year-old male with high-risk JAK2V617F-positive post-PV MF was initiated on ruxolitinib 10 mg twice daily to address worsening splenomegaly and debilitating fatigue. The patient experienced improvement in symptom burden and a decrease in palpable spleen size by 20%. However, he began to experience worsening ascites requiring large-volume paracentesis. Additionally, the ALP rose to 335 U/L from a baseline of around 180 U/L. Given the unknown cause of his liver dysfunction, he underwent a transjugular liver biopsy, demonstrating the presence of both EMH and OPV (Figure 1(C)). Because of these findings, the ruxolitinib dose was increased to 20 mg twice daily with improvement in symptoms and ascites and decrease in ALP to 151 U/L within 5 months.

### 2.4. Case 4

A 49-year-old male with DIPSS intermediate-2 risk, JAK2V617F-positive post-PV MF, and a history of portal vein thrombosis was started on ruxolitinib at 10 mg twice daily for splenomegaly. He attained an excellent symptomatic response but was noted to have an increase in total bilirubin to 2.6 mg/dL (44.5 *μ*mol/L) from a normal baseline. During this time, no new medications were started. Although some hyperbilirubinemia can be attributed to hemolysis, he underwent a liver biopsy which demonstrated OPV with extensive EMH. He was continued on ruxolitinib 15 mg with improvement in total bilirubin to 1.3 mg/dL (22.23 *μ*mol/L).

## 3. Discussion

There is a significant but incompletely understood relationship between MPNs and liver disease. Noncirrhotic portal hypertension (NCPH), a broad category of diseases including OPV, has been described in the setting of MPNs with the sequela including ascites, esophageal and gastric varices, and subsequent variceal bleeding. The development of NCPH is at least in part a consequence of thrombotic disease; however, inappropriate endothelial cell activation associated with *JAK2V617F* expression has been implicated [5]. OPV involves variable obliteration of the portal vein, particularly in the terminal branches of the intrahepatic portal vein, and has been observed in patients with MPNs [3]. Finally, EMH can manifest as a focal hepatic nodule [6], diffuse hepatomegaly and portal hypertension [7], or laboratory manifestations as in the first, second, and fourth cases described in the current series [8].

As demonstrated in a retrospective study of 398 PMF patients, the most common hepatic laboratory derangement was an elevated ALP, which was CTCAE grade 1 in 40% of patients, grade 2 in 7%, and grade 3 or above in 1%. When grade 2 or above, an elevated ALP was associated with a higher leukocyte count and worse prognosis. AST elevations were also present in this population, with 9% having elevations which were mostly grade 1. No findings were reported for other liver chemistry tests such as ALT or bilirubin levels [9]. The pathological basis of these laboratory findings was not reported, nor were concomitant medications including ruxolitinib noted.

The cases described in the current series illustrate the variety of liver pathology seen in PV/MF patients with liver dysfunction. However, it is unclear the prevalence of liver pathology in MPN patients without liver dysfunction. We also report a possible case of ruxolitinib-associated DILI. There are no known mechanisms to explain possible hepatotoxicity with this agent. Ruxolitinib is metabolized by the liver through the CYP3A4 system, with metabolites mainly excreted in the urine. It is plausible that liver injury may be related to the production of a toxic intermediate [10]. If DILI is suspected with ruxolitinib exposure, discontinuation may be warranted. However, abrupt discontinuation of ruxolitinib in any context can lead to a serious and potentially life-threatening withdrawal syndrome characterized by worsening cytopenias and progressive splenomegaly, the most severe form of which can mimic septic shock with hemodynamic instability [11]. Therefore, when ruxolitinib is discontinued, it should be done as a taper under close supervision and in certain cases overlapped with prednisone to blunt the potential cytokine rebound phenomenon. Thus, utilizing a liver biopsy to diagnose DILI is of paramount importance before considering withdrawal of ruxolitinib.

Although some liver abnormalities can be attributed to ruxolitinib itself, the majority of patients in our series had abnormalities as a consequence of EMH. This series emphasizes that obtaining a liver biopsy is a crucial and necessary step for adaptive management in patients with evidence of hepatocellular damage receiving ruxolitinib treatment. The liver biopsy may be instrumental in determining if ruxolitinib therapy should be discontinued or continued.
